# Plasma amino acids and metabolic profiling of dairy cows in response to a bolus duodenal infusion of leucine

**DOI:** 10.1371/journal.pone.0176647

**Published:** 2017-04-28

**Authors:** Hassan Sadri, Dirk von Soosten, Ulrich Meyer, Jeannette Kluess, Sven Dänicke, Behnam Saremi, Helga Sauerwein

**Affiliations:** 1 Institute of Animal Science, Physiology & Hygiene Unit, University of Bonn, Bonn, Germany; 2 Department of Clinical Science, Faculty of Veterinary Medicine, University of Tabriz, Tabriz, Iran; 3 Institute of Animal Nutrition, Friedrich-Loeffler-Institute (FLI), Braunschweig, Germany; 4 Evonik Nutrition & Care GmbH, Rodenbacher Chaussee 4, Hanau, Germany; Medical University of Vienna, AUSTRIA

## Abstract

Leucine (Leu), one of the three branch chain amino acids, acts as a signaling molecule in the regulation of overall amino acid (AA) and protein metabolism. Leucine is also considered to be a potent stimulus for the secretion of insulin from pancreatice β-cells. Our objective was to study the effects of a duodenal bolus infusion of Leu on insulin and glucagon secretion, on plasma AA concentrations, and to do a metabolomic profiling of dairy cows as compared to infusions with either glucose or saline. Six duodenum-fistulated Holstein cows were studied in a replicated 3 × 3 Latin square design with 3 periods of 7 days, in which the treatments were applied at the end of each period. The treatments were duodenal bolus infusions of Leu (DIL; 0.15 g/kg body weight), glucose (DIG; at Leu equimolar dosage) or saline (SAL). On the day of infusion, the treatments were duodenally infused after 5 h of fasting. Blood samples were collected at -15, 0, 10, 20, 30, 40, 50, 60, 75, 90, 120, 180, 210, 240 and 300 min relative to the start of infusion. Blood plasma was assayed for concentrations of insulin, glucagon, glucose and AA. The metabolome was also characterized in selected plasma samples (i.e. from 0, 50, and 120 min relative to the infusion). Body weight, feed intake, milk yield and milk composition were recorded throughout the experiment. The Leu infusion resulted in significant increases of Leu in plasma reaching 20 and 15-fold greater values than that in DIG and SAL, respectively. The elevation of plasma Leu concentrations after the infusion led to a significant decrease (*P*<0.05) in the plasma concentrations of isoleucine, valine, glycine, and alanine. In addition, the mean concentrations of lysine, methionine, phenylalanine, proline, serine, taurine, threonine, and asparagine across all time-points in plasma of DIL cows were reduced (*P*<0.05) compared with the other groups. In contrast to the working hypothesis about an insulinotropic effect of Leu, the circulating concentrations of insulin were not affected by Leu. In DIG, insulin and glucose concentrations peaked at 30–40 and 40–50 min after the infusion, respectively. Insulin concentrations were greater (*P*<0.05) from 30–40 min in DIG than DIL and SAL, and glucose was elevated in DIG over DIL and SAL from 30–75 min and 40–50 min, respectively. Multivariate metabolomics data analysis (principal component analysis and partial least squares discriminant analysis) revealed a clear separation when the DIL cows were compared with the DIG and SAL cows at 50 and 120 min after the infusion. By using this analysis, several metabolites, mainly acylcarnitines, methionine sulfoxide and components from the kynurenine pathway were identified as the most relevant for separating the treatment groups. These results suggest that Leu regulates the plasma concentrations of branched-chain AA, and other AA, apparently by stimulating their influx into the cells from the circulation. A single-dose duodenal infusion of Leu did not elicit an apparent insulin response, but affected multiple intermediary metabolic pathways including AA and energy metabolism by mechanisms yet to be elucidated.

## Introduction

The three branched-chain amino acids (BCAA), leucine (Leu), isoleucine (Ile), and valine (Val) that are classified as essential amino acids (AA), play important roles in regulating overall AA and protein metabolism. A body of evidence shows the key roles of BCAA, and Leu in particular, as signaling molecules in the regulation of protein synthesis [1,2]. Leucine supplementation in laboratory animal studies stimulated muscle protein synthesis during catabolic conditions associated with feed restriction or after exhaustive exercise [3,4]. Furthermore, among the AA, Leu is considered as the most effective in acting as a nutrient signal to modulate muscle protein synthesis and degradation in neonatal pigs [5,6]. Leucine has gained much attention as a potentially limiting AA for milk and milk protein yields in studies involving dairy cows [7–9]. Also, studies have shown that administration of BCAA (especially Leu) has a great impact on the plasma AA profiles in human [10,11], pig [12], and mice [13]. However, the physiological significance and the underlying molecular mechanisms responsible for the regulation of plasma AA profile after Leu administration are not yet well understood.

The postprandial increase of glucose in blood knowingly results in a release of insulin. Besides sugars such as glucose, other dietary substrates, e.g. protein and free AA can have strong insulinotropic effects, especially when co-ingested with carbohydrates. In humans and in laboratory animals, it has been demonstrated that Leu has an insulinotropic effect [14,15]. Leucine-induced insulin secretion is mediated by its oxidative decarboxylation as well as by allosteric activation of glutamate dehydrogenase [14]. However, studies regarding effects of BCAA feeding on glucose metabolism and insulin resistance have yielded contrasting results [15–17]. In contrast to the effects of glucose and free fatty acid, the effects of AA, the third class of nutrients as stimulus of insulin secretion have scarcely been investigated in dairy cows. To what extent Leu may also affect the secretion of insulin in dairy cows has not yet been investigated. However, in view of the metabolic situation in dairy cows entering lactation, increasing the release of insulin might be an interesting option to support the metabolic adaptation to the rapidly increasing milk production. Similar to other mammals, dairy cows develop insulin resistance during pregnancy and early lactation to facilitate the drain of glucose to the fetus and the mammary gland. The insulin resistance comprises both reduced insulin secretion and reduced insulin sensitivity of peripheral organs [18–20].

We hypothesized that Leu stimulates the release of insulin in dairy cows, and thus the objective was to characterize the response of insulin, glucagon, glucose, and AA in blood plasma towards Leu as compared to infusions with either glucose or saline. To test this hypothesis, an animal experiment was performed with duodenum-fistulated cows in order to circumvent degradation of Leu by the ruminal microflora. Also, the targeted metabolomics approach was used to gain deeper views into the actual metabolic and physiological state of the animals post-infusion.

## Materials and methods

### Experimental design and treatments

The experiment was conducted at the experimental station of the Friedrich-Loeffler-Institute (Braunschweig, Germany). The experimental procedures performed in this study were in accordance with the regulations of the European Community concerning the protection of experimental animals and were approved by the Lower Saxony State Office for Consumer Protection and Food Safety, Germany (LAVES). Six pluriparous, duodenum-fistulated German Holstein cows (BW = 652 ± 29.2 kg; mean ± SE) in late lactation (days in milk = 289 ± 11.3; mean ± SEM), were studied in a replicated 3 × 3 Latin square design with 3 periods of 7 days, in which the treatments were applied at the end of each period. The animals were fixed in the head locker after a defined period of fasting (5 h), fitted with permanent venous catheters and the infusions were then applied through the duodenum fistulae. All husbandry set-up and procedures were in accordance with good agricultural practice and were in consideration of the respective legal provisions. In addition, all procedures involved in this study were minimally invasive and did not involve any pain, suffering, distress or lasting harm. Animals were monitored for any disorders by the farm veterinarians during the course of the study. There were no signs for any health disturbances during the course of the study. Animals remained in the dairy herd of the experimental station of the Friedrich-Loeffler-Institute (Braunschweig, Germany) after the end of this study. The treatments were single bolus infusions of either Leu [**DIL**; 0.15 g/kg body weight (BW); with a molarity of 0.8 M which corresponds to 106 g Leu per liter; Evonik Nutrition & Care GmbH, Hanau, Germany, Pharma level], glucose (**DIG**; with the same molar amount as that of Leu; i.e. 0.8 M glucose which corresponds to 145.6 g glucose per liter; Sigma-Aldrich, Taufkirchen, Germany) and saline [**SAL**; 0.9% NaCl (w/v; Oxoid Ltd., Basingstoke, UK) prepared using distilled/deionized water]. Due to the low solubility of Leu (24.3 g/L, [21,22]), the infusate was used as suspension rather than a true solution. The total volume of the infusate was between 0.94 and 1.23 L. The dosage calculation was based on typical glucose tolerance test that is performed in cattle using a single intravenous bolus infusion with 0.15 g glucose/kg body weight [23,24]. The calculated dosage, i.e., 0.15 g/kg BW was considered for Leu, and in the case of glucose, the same molarity as that of Leu was used. During the experimental period, the cows received a feed ration formulated to meet the nutritional requirements of the cows stated by the German Society of Nutrition Physiology [25]. Animals were fed a partial mixed ration (PMR) for *ad libitum* consumption consisting of 20% concentrate and 80% silage (40% maize silage, 40% grass silage based on dry matter content). Additionally, cows received 4 kg concentrate per day via an automatic concentrate feeder. The concentrate, which was included in the PMR, consisted of the same components as the concentrate provided by the automatic concentrate feeder. The concentrate contained wheat (39.5%), dried sugar beet pulp (29.7%), rapeseed meal (20.5%), soybean meal (6.70%), soybean oil (1%), calcium carbonate (0.5%), and mineral feed (2.10%). Body weights were recorded daily throughout the study.

### Feed and milk sampling and analyses

The amounts of feed offered and orts were recorded daily for individual cows and daily dry matter intake (DMI) was determined. Forage, concentrate, and PMR were sampled weekly during the experiment and stored at -20°C. Feed samples were dried at 60°C for 72 h, ground to pass through a 1-mm pore size and stored at −20°C until analysis. Feed samples were analyzed for crude protein, ether extract, crude fiber, acid detergent fiber, neutral detergent fiber, and total ash according to the methods of the VDLUFA [26]. Feed samples were also analyzed for AA by Evonik Nutrition & Care GmbH using ion-exchange chromatography with postcolumn derivatization with ninhydrin [27,28]. Amino acids were oxidized with performic acid, which was neutralized with Na metabisulfite. Amino acids were liberated from the protein by hydrolysis with 6 N HCL for 24 h at 110°C and quantified with the internal standard by measuring the absorption of reaction products with ninhydrin at 570 nm. Tryptophan was determined by HPLC with fluorescence detection (extinction 280 nm, emission 356 nm), after alkaline hydrolysis with barium hydroxide octahydrate for 20 h at 110°C. Cows were milked 2 times daily and yields were recorded at each milking. Milk samples were collected weekly from 2 consecutive milkings and preserved using bronopol and stored at 8°C until analyzed. Milk samples were analyzed for fat, protein, and lactose using an infrared milk analyser (Milkoscan FT 6000 combined with a Fossomatic 500, Foss Electric, Hillerød, Denmark).

### Blood sampling and analyses

The cows were fitted with catheters (WVI Jugularis-Katheter, OD 2.4 mm, length 20 cm incl. Mandrin, Walter Veterinärinstrumente e.K., Baruth/Mark, Germany) in the jugular vein approximately 12 h before the start of infusion. Blood samples were taken at -15, 0, 10, 20, 30, 40, 50, 60, 75, 90, 120, 180, 210, 240 and 300 min relative to the infusion and were immediately centrifuged (1,500 × *g*, 15 min at 4°C) and plasma was stored at -20°C until analyzed. Glucose was measured in sodium fluoride-EDTA plasma at synlab.vet GmbH, Labor Leverkusen (Leverkusen, Germany) using a photometric assay. Plasma concentrations of insulin were determined using a bovine insulin ELISA kit (Mercodia Bovine Insulin ELISA; Mercodia AB, Uppsala, Sweden) with intra- and inter-assay coefficients of variation 2.9% and 3.2%, respectively. Plasma concentrations of glucagon were measured using the glucagon chemiluminescent ELISA kit (EMD Millipore Corporation, Billerica, MA, USA) with intra- and inter-assay coefficients of variation 5.1% and 6.2%, respectively. The spectrophotometric measurements were conducted using a microplate reader (Synergy H1, BioTek, Winooski, VT, USA). Plasma free AA concentrations were determined by Evonik via ion exchange chromatography using a Biochrom 20 amino acid analyzer with lithium column and lithium buffers [27,28]. The samples were deproteinized using sulfosalicylic acid. The metabolome was characterized in blood plasma samples collected at 0, 50, and 120 min relative to the infusion using a targeted quantitative metabolomics approach employing a commercially available Kit (AbsoluteIDQ p180 Kit; Biocrates Life Sciences AG, Innsbruck, Austria). The kit plates were used for the quantification of 186 different metabolites including 21 AA, 40 acylcarnitines, 15 sphingomyelins, 90 glycerophospholipids, 19 biogenic amines and hexoses. The fully automated assay was based on PITC (phenylisothiocyanate)-derivatization in the presence of internal standards followed by FIA-MS/MS (acylcarnitines, lipids, and hexose) and LC/MS (amino acids, biogenic amines) using an AB SCIEX 4000 QTrap mass spectrometer (AB SCIEX, Darmstadt, Germany) with electrospray ionization. The experimental metabolomics measurement technique was already described in detail [29].

### Statistical analysis

The adaptation period values (the first 2 week of the experiment) for DMI, BW, and milk yield and milk composition were used as subject-dependent covariates which were constant over the repeated measures. Performance data were analyzed with the PROC MIXED of SAS (version 9.2). The effects of treatment, square, and period were fixed and the effect of cow was random. In addition, the covariates were also considered as fixed effects.

Blood plasma glucose, insulin, glucagon, and AA data were analyzed using repeated measure in the MIXED procedure of SAS. The model included square, period (within square), treatment, time (sampling min), and interaction of treatment and time as the fixed effects and cow (within square) as the random effect. The compound symmetry was used for the covariance structure. The Tukey-Kramer adjustment was used to account for multiple comparisons. The threshold of significance was set at *P*≤0.05; trends were declared at 0.05<*P*<0.10. Data are presented as means ± SEM.

Statistical analyses of the metabolomics data were performed using MetaboAnalyst 3.0 according to previously published protocols [30,31]. The metabolomics data were cleaned up for any removable noise or any missing values to be imputed and then were processed by log transformation and Pareto scaling prior to the statistical analysis. Univariate analysis including fold-change analysis, t-test, and volcano plots were first performed in order to assess the differences of detected metabolites between the groups and to obtain an overview and rough ranking of potentially important metabolites. Volcano plots are used to relate fold-change to the statistical significance level. Furthermore, principal component analysis (PCA), partial least squares discriminant analysis (PLS-DA), and variable importance of projection (VIP) were performed to identify those metabolites showing significant differences among the treatment groups. PCA, an un-supervised method, is used to find the direction of maximum variance in a complex collection of data. PLS-DA, a supervised method, can perform classification and feature selection. Furthermore, a VIP plot is used to rank the metabolites based on their importance in discriminating treatment groups.

## Results

### Performance data

Table 1 shows BW, DMI, milk yield, fat corrected milk (FCM), and milk composition in dairy cows during the experimental period. Neither variable was different between the treatments, except in the case of PMR DMI, which was greater in DIG than DIL (*P* = 0.007). Crude protein and AA contents of the forages, concentrates, and PMR used during the experiment are shown in the S1 Table.

**Table 1:** Body weight (BW), dry matter intake (DMI), milk yield, fat corrected milk (FCM) and milk composition in dairy cows during the experimental period.

	Treatments^1^			Treatment effect (*P*-value)
Item	DIG	DIL	SAL	
BW, kg	651 ± 30	650 ± 28	655 ± 29	0.18
DMI, kg/d				
PMR^2^	11.2 ± 0.7^a^	10.7 ± 0.4^b^	10.9 ± 0.8^a^^b^	0.007
Concentrate	3.1 ± 0.2	3.1 ±0.2	3.1 ± 0.2	0.78
Milk yield, kg/d	16.2 ± 1.5	16.1 ± 1.6	16.1 ± 1.5	0.94
FCM,^3^ kg/d [63]	16.3 ± 1.1	15.9 ± 1.4	16.4 ± 1.4	0.42
Milk composition				
Fat, %	4.20 ± 0.34	4.03 ± 0.25	4.09 ± 0.22	0.25
Fat, kg/d	0.67 ± 0.05	0.64 ± 0.06	0.65 ± 0.05	0.47
Protein, %	3.41 ± 0.06	3.35 ± 0.06	3.37 ± 0.06	0.34
Protein, kg/d	0.55 ± 0.04	0.54 ± 0.05	0.54 ± 0.04	0.74
Lactose, %	4.61 ± 0.09	4.57 ± 0.09	4.60 ± 0.09	0.70
Lactose, kg/d	0.75 ± 0.07	0.74 ± 0.08	0.74 ± 0.07	0.78

^1^Single-dose duodenal infusions of glucose (DIG), leucine (DIL), or saline (SAL).

^2^Partial mixed ration.

^3^FCM was estimated with the following formula: FCM [kg/d] = ((milk fat [%] * 0.15) + (0.4 * milk yield [kg/d]).

^a^ Different superscripts within a row indicate significance at *P*<0.05.

^b^Different superscripts within a row indicate significance at *P*<0.05.

### Plasma AA, glucose, insulin, and glucagon concentrations

**Fig 1:**
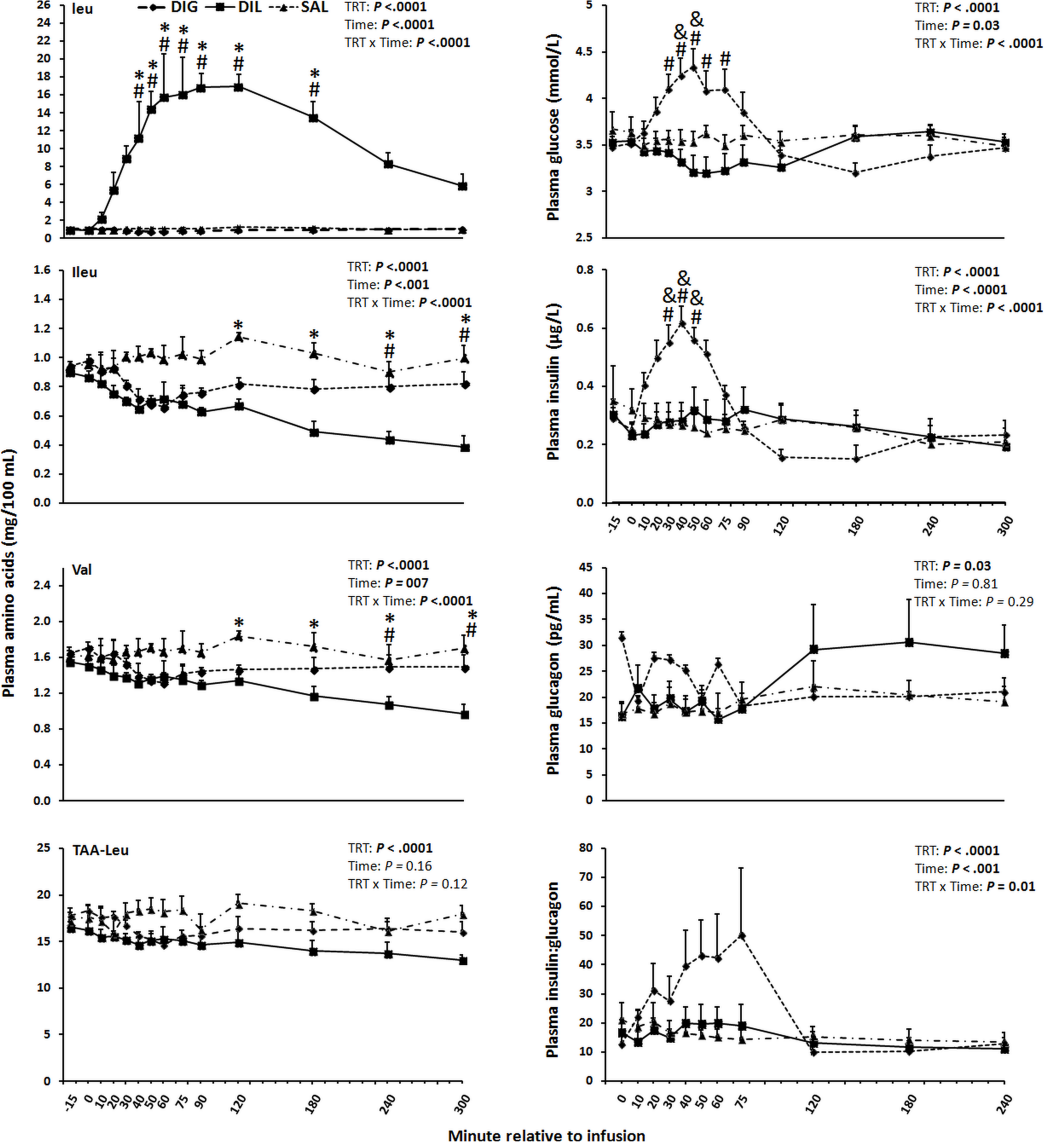
Plasma concentrations of branched-chain amino acids (Leucine, Ileucine, Valine), total amino acids minus leucine (TAA-Leu), glucose, insulin, glucagon, and insulin:glucagon in response to single-dose duodenal infusions of glucose (DIG), leucine (DIL), or saline (SAL) in dairy cows. Within minute, symbols indicate the following treatment differences (*P*<0.05); # = DIL vs. DIG, * = DIL vs. SAL, & = DIG vs. SAL. TRT = treatment.

As shown in Fig 1, the effects of treatment, time, and the interaction of treatment × time were significant for the concentrations of Leu in plasma (*P*<0.0001). Leu in plasma of DIL cows peaked at 50–60 min after the infusion and was 20 and 15-fold greater than that in DIG and SAL, respectively. Furthermore, from 40–180 min the Leu concentrations in DIL were greater (*P*≤0.005) than in DIG and SAL. Plasma glucose concentrations were affected by treatment, time, and interaction between treatment and time (*P*≤0.03; Fig 1). In DIG cows, the glucose concentrations peaked 40–50 min after the infusion and were greater (*P*≤0.01) from 30–75 and 40–50 min than in the DIL and SAL treatments, respectively. Plasma insulin concentrations were affected by treatment, time, and interaction between treatment and time (*P*<0.0001). In DIG, insulin concentrations peaked 30–40 min after the infusion. Insulin concentrations were greater (*P*<0.05) from 30–50 min in DIG than DIL and SAL. No differences between DIL and SAL groups were observed over time for plasma insulin concentrations. The plasma concentrations of glucagon were neither affected by time, nor treatment by time interaction, whereas the overall treatment effect was significant (*P* = 0.03; Fig 1). The mean concentrations of glucagon across all time-points in DIG were greater than in SAL (*P* = 0.02; Fig 1). Plasma insulin: glucagon was affected by treatment, time, and interaction between treatment and time (*P*≤0.01). Plasma insulin: glucagon across all time-points in DIG was greater than in the other groups (*P*≤0.0001; Fig 1).

**Fig 2:**
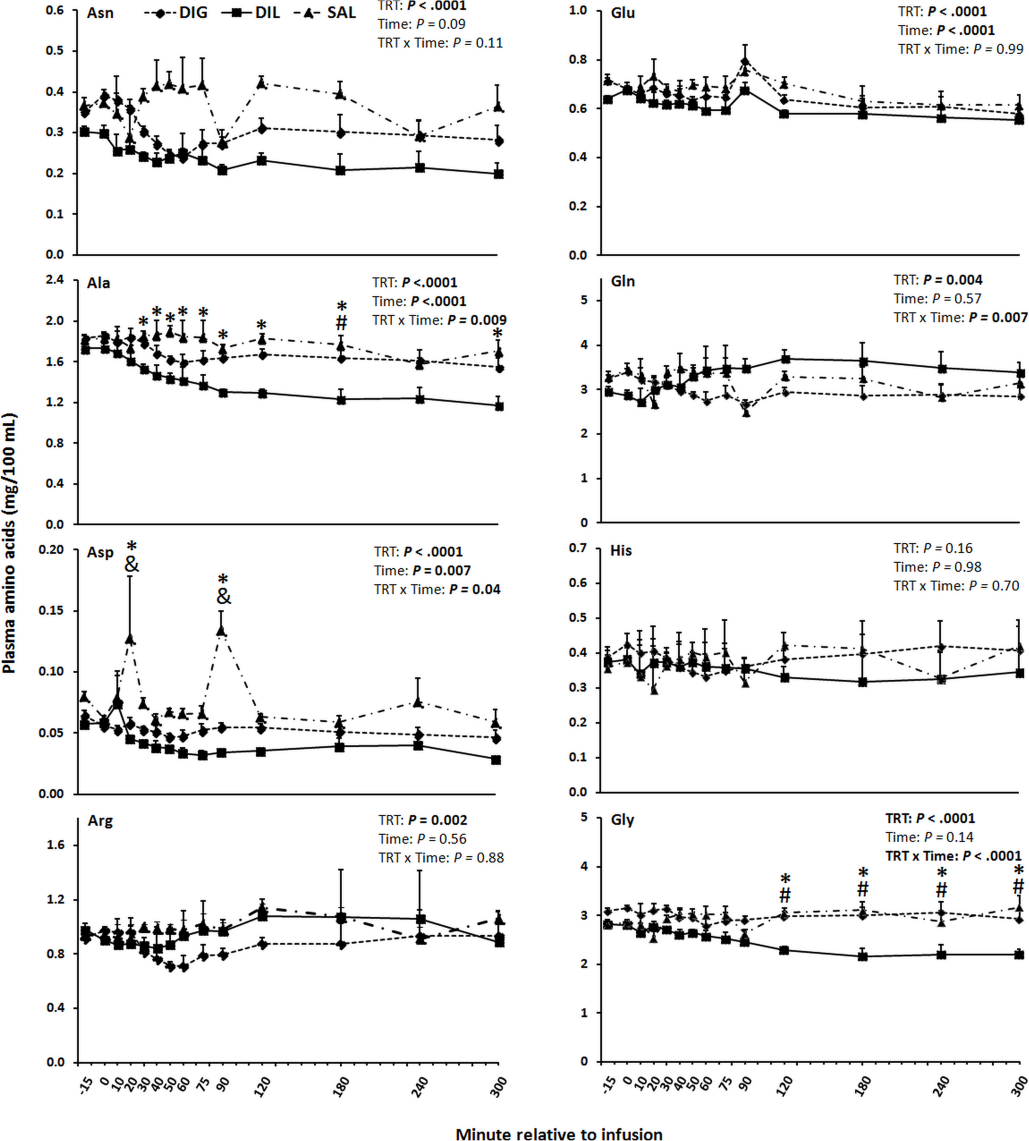
Plasma concentrations of Asn, Ala, Asp, Arg, Glu, Gln, His, and Gly in response to single-dose duodenal infusions of glucose (DIG), leucine (DIL), or saline (SAL) in dairy cows. Within minute, symbols indicate the following treatment differences (*P*<0.05); # = DIL vs. DIG, * = DIL vs. SAL, & = DIG vs. SAL. TRT = treatment.

**Fig 3:**
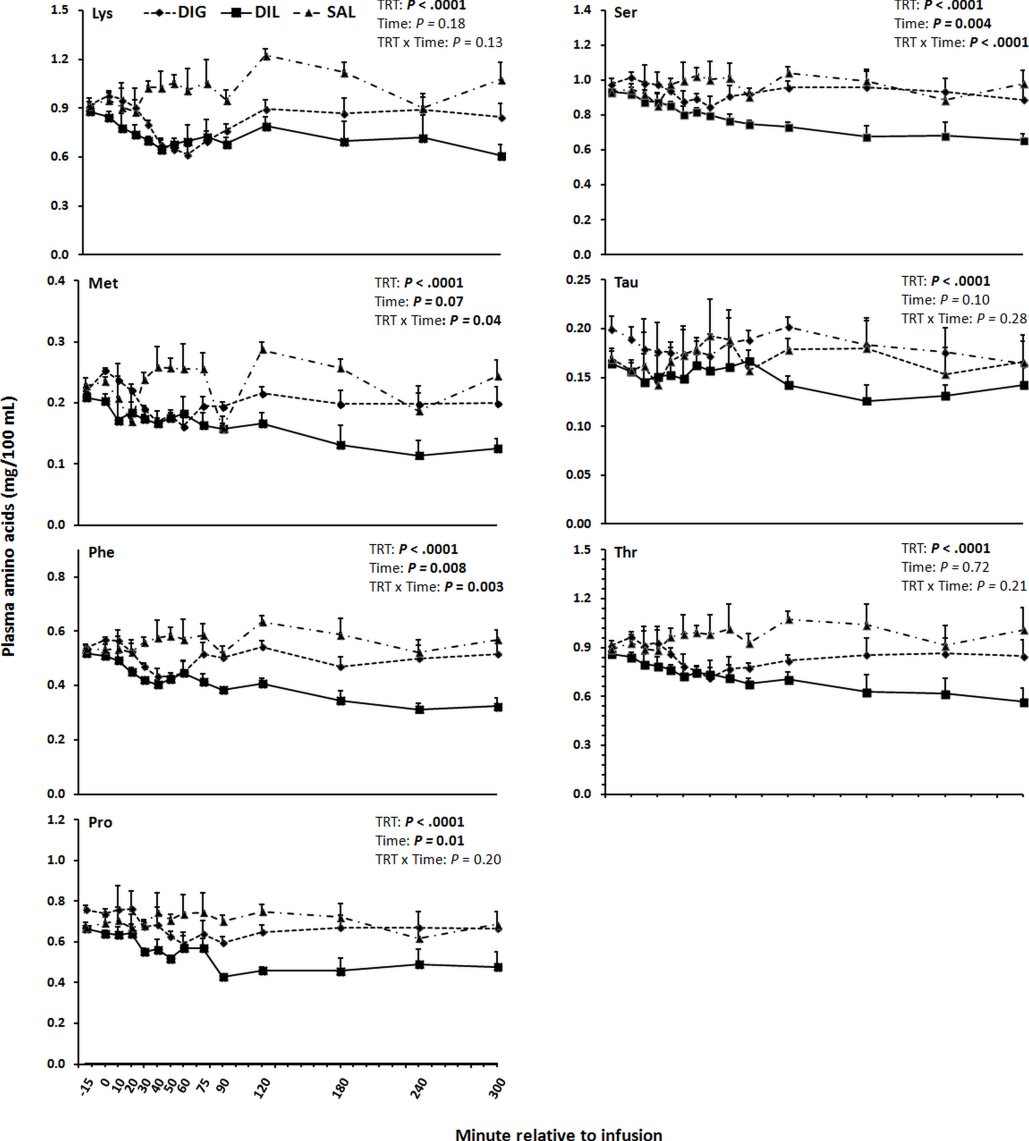
Plasma concentrations of Lys, Met, Phe, Pro, Ser, Tau, and Thr in response to single-dose duodenal infusions of glucose (DIG), leucine (DIL), or saline (SAL) in dairy cows.

In DIL cows, the plasma concentration of isoleucine (Ile) and valine (Val) were lower (*P*≤0.01) from 120–300 and 240–300 min than in the SAL and DIG cows, respectively (Fig 1). The plasma concentrations of total AA minus Leu were neither affected by time, nor treatment by time interaction, whereas the overall treatment effect was significant (*P*<0.0001; Fig 1). The lowest mean concentrations of total AA minus Leu were observed in DIL, followed by DIG and the highest in SAL. The plasma concentrations of alanine (Ala) starting from 40 min after the infusion and of glycine (Gly) starting from 120 min after the infusion were lower in DIL cows than in SAL and DIG cows (*P*<0.001; Fig 2). In addition, mean concentrations of asparagine (Asn), lysine (Lys), methionine (Met), phenylalanine (Phe), proline (Pro), serine (Ser), taurine (Tau), and threonine (Thr) across all time-points in DIL were lower than in the other groups (*P*≤0.02; Figs 2 and 3). Plasma concentrations of histidine (His) were neither affected by treatment, time, nor treatment by time interaction (Fig 2).

### Metabolomics data

#### Univariate statistical analysis

The univariate analyses including t-tests, and volcano plot were performed to obtain a preliminary overview about the metabolites that are contributing most significantly in discriminating the groups. Tables 2–4 show the results of significantly (*P*≤0.05) changed metabolites identified by t-tests in the treatment groups at 50 and 120 min after the infusion. S1–S3 Figs also show the significant metabolites identified by volcano plot at 50 and 120 min after the infusion in the treatment groups. The volcano plot shows the relationship between the *P*-values of the statistical test and the magnitude of the difference in concentrations between the treatment groups. The x-axis displays the mean of log2 fold-change value, and the y-axis corresponds to the negative logarithm of the *P*-values. Each circle represents one metabolite. The dashed horizontal line shows the level of significance for the t-tests performed (0.10), and the vertical dashed lines indicate the threshold set for fold-change (1.3). The red circles represent significantly changed metabolites; the grey circles did not reach significance. As expected, most of the significant metabolites identified by volcano plot are similar to those of the t-test.

**Table 2:** Significant plasma metabolites (mean ± SE) identified by t-test at 50 and 120 min after duodenal bolus infusions of leucine (DIL) as compared to glucose (DIG) in dairy cows.

	Treatments			
Metabolites, μM^1^	DIL	DIG		*P*-value
**50 min**				
H1	3093.7 ± 140.1		4169.3 ± 213.1	0.001
Leucine	1682.7 ± 604.8		67.0 ± 9.15	0.002
Kynurenine	4.38 ± 0.43		6.99 ± 0.52	0.004
**120 min**				
Leucine	1877.7 ± 478.4		85.1 ± 10.1	<0.0001
PC aa C42:0	0.63 ± 0.02		0.64 ± 0.02	<0.01
Ileucine	118.0 ± 18.7		63.7 ± 6.09	0.01
Kynurenine	3.89 ± 0.42		5.81 ± 0.49	0.01
C2	1.09 ± 0.16		0.65 ± 0.11	0.02
C12	0.03 ± 0.001		0.02 ± 0.0007	0.03
C6/C4:1-DC	0.026 ± 0.002		0.021 ± 0.0006	0.03
C7-DC	0.017 ± 0.0009		0.015 ± 0.0006	0.03
C18:1-OH	0.0080 ± 0.0003		0.0092 ± 0.0004	0.04
PC aa C24:0	0.071 ± 0.007		0.049 ± 0.006	0.05
Alanine	147 ± 8.84		190.7 ± 16.9	0.05

^1^H1 = Glucose, Aldohexose, L-Allopyranose, D-Allose,…; PC aa C42:0 = Phosphatidylcholine with diacyl residue sum C42:0; C2 = Acetylcarnitine; C12: Dodecanoylcarnitine [= Laurylcarnitine]; C6/C4:1-DC = Hexanoylcarnitine [= Caproylcarnitine] (Fumarylcarnitine); C7-DC = Pimelylcarnitine; C18:1-OH = Hydroxyoctadecenoylcarnitine [= Hydroxyoleylcarnitine]; PC aa C24:0 = Phosphatidylcholine with diacyl residue sum C24:0.

**Table 3:** Significant plasma metabolites (mean ± SE) identified by t-test at 50 and 120 min after duodenal bolus infusions of leucine (DIL) as compared to saline (SAL) in dairy cows.

	Treatments		
Metabolites, μM^1^	DIL	SAL	*P*-value
**50 min**			
Leucine	3093.7 ± 140.1	95.5 ± 6.4	0.005
C16-OH	0.005 ± 0.0004	0.003 ± 0.0002	0.02
Proline	45.8 ± 4.49	67.4 ± 8.07	0.03
Alanine	162.7 ± 12.9	212 ± 16.2	0.03
C3-OH	0.02 ± 0.002	0.01 ± 0.001	0.05
C3-DC-M/C5-OH	0.06 ± 0.004	0.05 ± 0.001	0.05
**120 min**			
Leucine	1877.7 ± 478.4	113.1 ± 9.32	<0.0001
Alanine	147 ± 8.84	210.2 ± 14.3	0.003
Kynurenine	3.89 ± 0.42	5.94 ± 0.25	0.003
C5	0.05 ± 0.007	0.03 ± 0.002	0.02
Proline	44 ± 4.42	62.8 ± 5.16	0.02
C18:1-OH	0.008 ± 0.0003	0.009 ± 0.0004	0.02
Lysin	58.2 ± 8.66	92.2 ± 10.8	0.03
Asparagine	20.3 ± 3.25	33.4 ± 3.73	0.03
Tyrosin	25 ± 5.18	42.2 ± 5.44	0.04

^1^C16-OH = Hydroxyhexadecanolycarnitine [= Hydroxypalmitoylcarnitine]; C3-OH = Hydroxypropionylcarnitine; C3-DC-M/C5-OH = Hydroxyisovalerylcarnitine / Hydroxy-2-methylbutyryl / Hydroxyvalerylcarnitine (Methylmalonylcarnitine); C5 = Isovalerylcarnitine / 2-Methylbutyrylcarnitine / Valerylcarnitine; C18:1-OH = Hydroxyoctadecenoylcarnitine [= Hydroxyoleylcarnitine].

**Table 4:** Significant plasma metabolites (mean ± SE) identified by t-test at 50 and 120 min after duodenal bolus infusions of glucose (DIG) as compared to saline (SAL) in dairy cows.

	Treatments			
Metabolites, μM^1^	DIG	SAL		*P*-value
**50 min**				
H1	4169.3 ± 213.1		3235.7 ± 75.5	0.001
C3-DC-M / C5-OH	0.053 ± 0.002		0.046 ± 0.001	0.008
C12	0.024 ± 0.001		0.021± 0.001	0.01
Tyrosine	22.7 ± 1.75		38.5 ± 5.72	0.01
C18:1-OH	0.01 ± 0.0007		0.007 ± 0.0003	0.02
Isoleucine	58.3 ± 5.58		79.6 ± 6.08	0.03
Lysine	49.8 ± 4.58		74.7 ± 10.2	0.03
Ornithine	31.4 ± 2.52		41.1 ± 3.18	0.03
Leucine	70 ± 9.15		95.5 ± 6.40	0.04
C14	0.02 ± 0.001		0.01 ± 0.001	0.04
**120 min**				
Isoleucine	63.7 ± 6.09		95.6 ± 9.01	0.02
C7-DC	0.015 ± 0.001		0.017 ± 0.001	0.03
Lysine	60.7 ± 8.87		92.2 ± 10.8	0.04

^1^H1 = Glucose, Aldohexose, L-Allopyranose, D-Allose,…; C3-DC-M / C5-OH = Hydroxyisovalerylcarnitine / Hydroxy-2-methylbutyryl / Hydroxyvalerylcarnitine (Methylmalonylcarnitine); C12 = Dodecanoylcarnitine [= Laurylcarnitine]; C18:1-OH = Hydroxyoctadecenoylcarnitine [= Hydroxyoleylcarnitine]; C14 = Tetradecanoylcarnitine [= Myristylcarnitine]; C7-DC = Pimelylcarnitine.

The plasma concentrations of Leu increased (*P* = 0.002), whereas that of hexose (H1) and kynurenine decreased (*P*≤0.004) in the DIL cows as compared with the DIG cows at 50 min after the infusion. At 120 min after the infusion, the plasma concentrations of Leu, Ile, acetylcarnitine (C2), laurylcarnitine (C12), fumarylcarnitine (C6/C4:1-DC), pimelylcarnitine (C7-DC), and phosphatidylcholine with diacyl residue sum C24:0 (PC aa C24:0) increased (*P*≤0.05), whereas those of phosphatidylcholine with diacyl residue sum C42:0 (PC aa C42:0), kynurenine, hydroxyoleylcarnitine (C18:1-OH), and Ala were decreased (*P*≤0.05) in DIL compared to DIG (Table 2).

At 50 min after the infusion, plasma Leu, hydroxyhexadecanolycarnitine (C16-OH), hydroxypropionylcarnitine (C3-OH), and hydroxyisovalerylcarnitine/hydroxy-2-methylbutyryl/hydroxyvalerylcarnitine (C3-DC-M/C5-OH) concentrations were greater (*P*≤0.05), whereas that of Pro and Ala were lower (*P* = 0.03) in DIL than in DIG (Table 3). The plasma concentrations of Leu and isovalerylcarnitine/2-Methylbutyrylcarnitine/valerylcarnitine (C5) were elevated (*P*≤0.02), whereas that of Ala, kynurenine, Pro, C18:1-OH, Lys, Asp, and tyrosine (Tyr) were decreased (*P* = 0.04) in the DIL cows as compared with the SAL cows at 120 min after the infusion (Table 3).

The plasma concentrations of H1, C3-DC-M/C5-OH, C12, C18:1-OH, and myristylcarnitine (C14) increased (*P*≤0.04), whereas that of Tyr, Ile, Lys, ornithine (Orn), and Leu decreased (*P*≤0.04) in the DIG cows as compared with the SAL cows at 50 min after the infusion (Table 4). At 120 min after the infusion, plasma Ile, C7-DC, and Lys concentrations were lower (*P*≤0.04) in DIG than in SAL (Table 4).

#### Multivariate analysis on plasma metabolites at 50 min after the infusion

**Fig 4:**
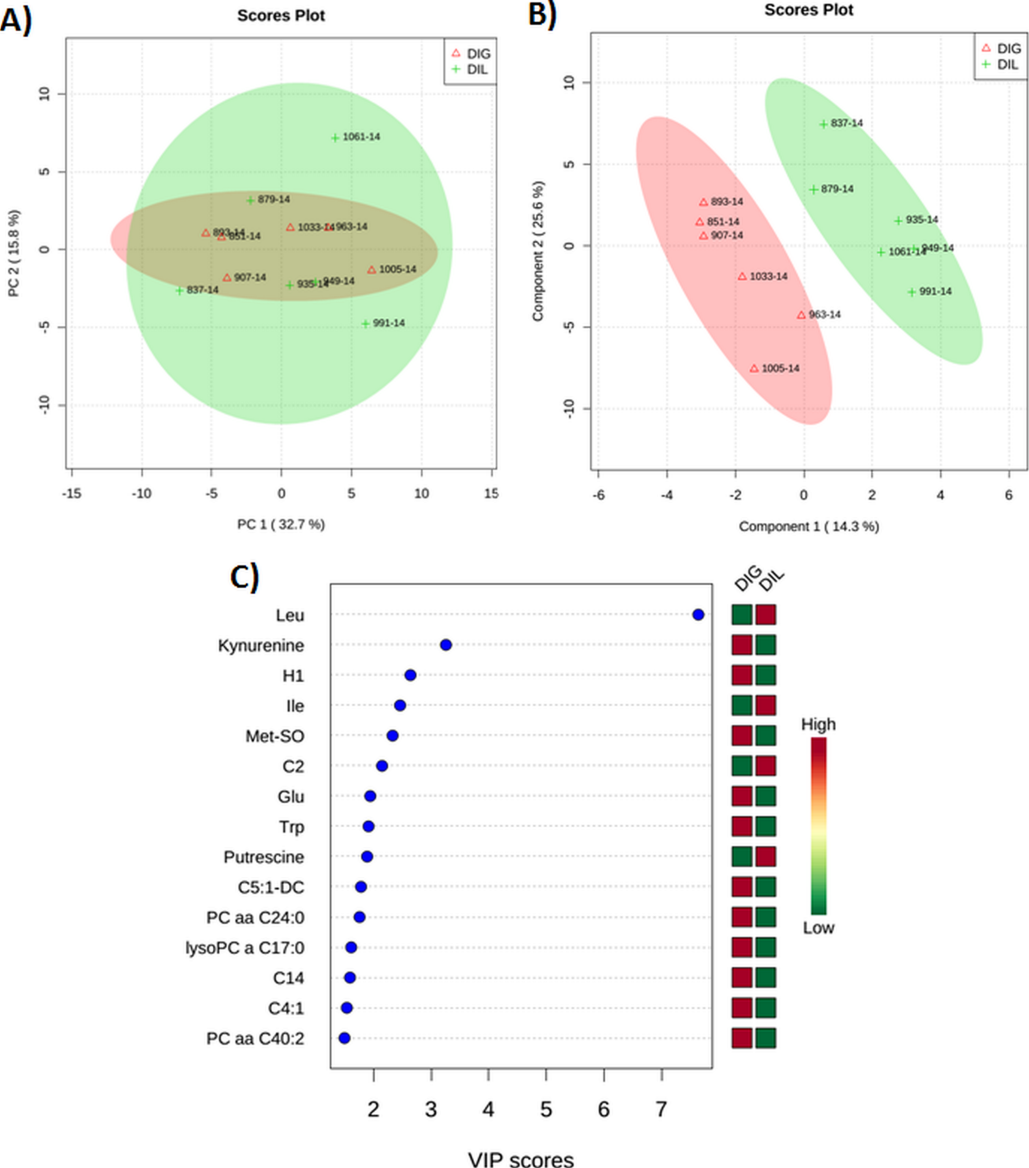
**(A) Score plot of principal component (PC) analysis of dairy cows at 50 min after duodenal bolus infusions of leucine (DIL) as compared with glucose (DIG). (B) Partial least squares-discriminant analysis showing 2 clusters for DIL and DIG groups and (C) metabolites ranked by variable importance in projection (VIP).** The numbers in the score plots represent the code numbers of the individual animals.

**Fig 5:**
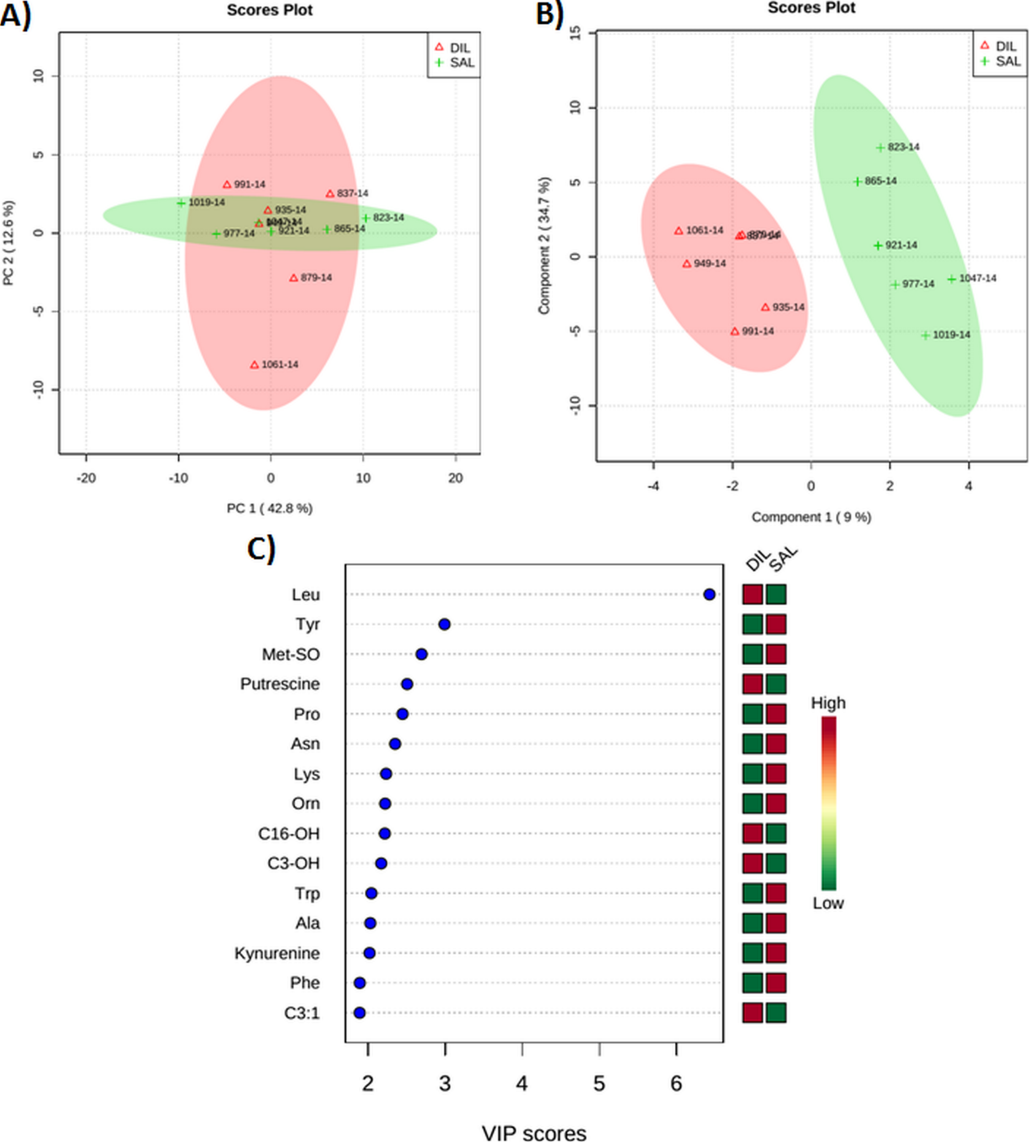
**(A) Score plot of principal component (PC) analysis of dairy cows at 50 min after duodenal bolus infusions of leucine (DIL) as compared with saline (SAL). (B) Partial least squares-discriminant analysis showing 2 clusters for DIL and SAL groups and (C) metabolites ranked by variable importance in projection (VIP).** The numbers in the score plots represent the code numbers of the individual animals.

Figs 4 and 5 show PCA and PLS-DA score plots and VIP plots in the DIL cows as compared with the DIG or SAL cows at 50 min after the infusion. The score plots were used to visualize sample clustering patterns by projection of the data onto the principal components in a way that explains the maximal variance (PCA) or co-variance (PLS-DA) of the data [31]. The VIP estimates the importance of each variable in the projection used in the PLS-DA model and thus driving the observed separation between groups. The VIP score of a variable is calculated as a weighted sum of the squared correlations between the PLS-DA components and the original variable [31]. The X-axis indicates the VIP scores corresponding to each variable on the Y-axis. When DIL cows were compared with DIG cows at 50 min after the infusion, 2 clear clusters could be seen (3A and B). Furthermore, 5 plasma metabolites including Leu, kynurenine, H1, Ile, methionine sulfoxide (Met-SO) with VIP scores of 7.6, 3.3, 2.6, 2.5, and 2.3, respectively, contributed most significantly to the separation between the DIL and DIG groups (Fig 4C). When DIL cows were compared with SAL cows at 50 min after the infusion, the multivariate analysis revealed a separation between the 2 groups (Fig 5A and 5B). Five metabolites including Leu, Tyr, Met-SO, putrescine, and Pro with corresponding VIP scores of 3.4, 3, 2.7, 2.5, and 2.5 contributed most significantly to the observed separation (Fig 5C). In this case, two compounds (Leu and putrescine) were elevated, whereas 3 metabolites (Tyr, Met-SO, and Pro) were decreased in the DIL cows as compared with the SAL cows. Interestingly, Met-SO is the same plasma metabolite responsible for separating the DIL and DIG groups at 50 min after the infusion.

When DIG cows were compared with SAL cows at 50 min after the infusion, the PCA and PLS-DA plots showed a separation between the 2 groups (S4A and S4B Fig). In this case, 5 blood plasma metabolites including Tyr, Lys, Ile, Asn, and H1 with VIP scores of 3.3, 2.8, 2.6, 2.5, and 2.5 contributed most significantly to the separation between the 2 groups (S4C Fig). All of these metabolites, except H1 were decreased in the DIG cows as compared with the SAL cows.

#### Multivariate analysis on plasma metabolites at 120 min after the infusion

**Fig 6:**
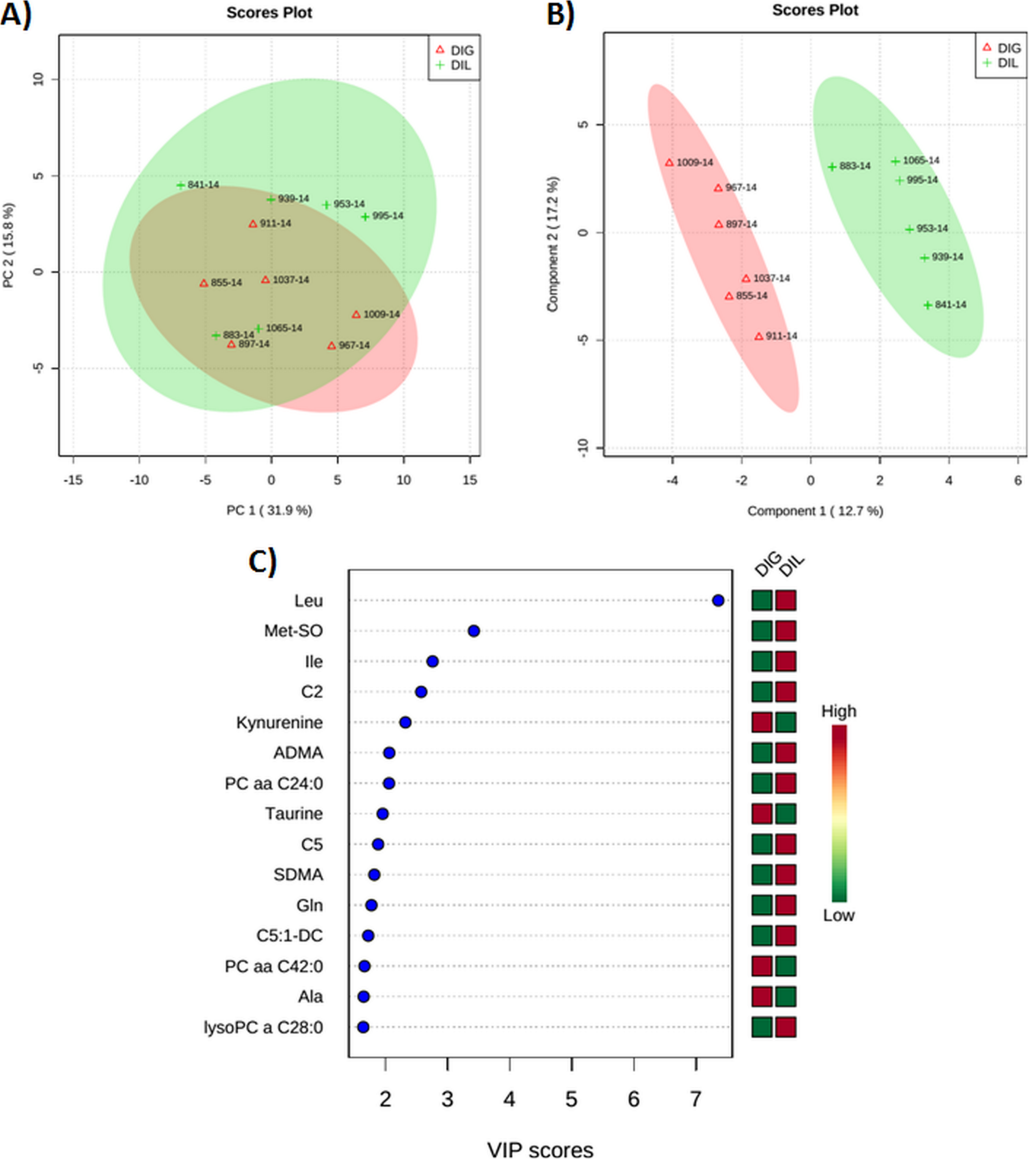
**(A) Score plot of principal component (PC) analysis of dairy cows at 120 min after duodenal bolus infusions of leucine (DIL) as compared with glucose (DIG). (B) Partial least squares-discriminant analysis showing 2 clusters for DIL and DIG groups and (C) metabolites ranked by variable importance in projection (VIP).** The numbers in the score plots represent the code numbers of the individual animals.

When DIL cows were compared with DIG cows at 120 min after the infusion, PCA and PLS-DA once again revealed a clear separation between the 2 groups (Fig 6A and 6B). Furthermore, 5 metabolites accounted for most of the observed separation, Leu, Met-SO, Ile, C2, and kynurenine with VIP scores of 7.4, 3.4, 2.8, 2.6, and 2.3, respectively (Fig 6C). The 4 metabolites (Leu, Met-SO, Ile, and kynurenine) most responsible for the separation of the DIL and DIG groups at 50 and 120 min after the infusion were similar. However, it is interesting to note that at 50 and 120 min, plasma Met-SO changed inversely in the DIL cows as compared with the DIG cows.

**Fig 7:**
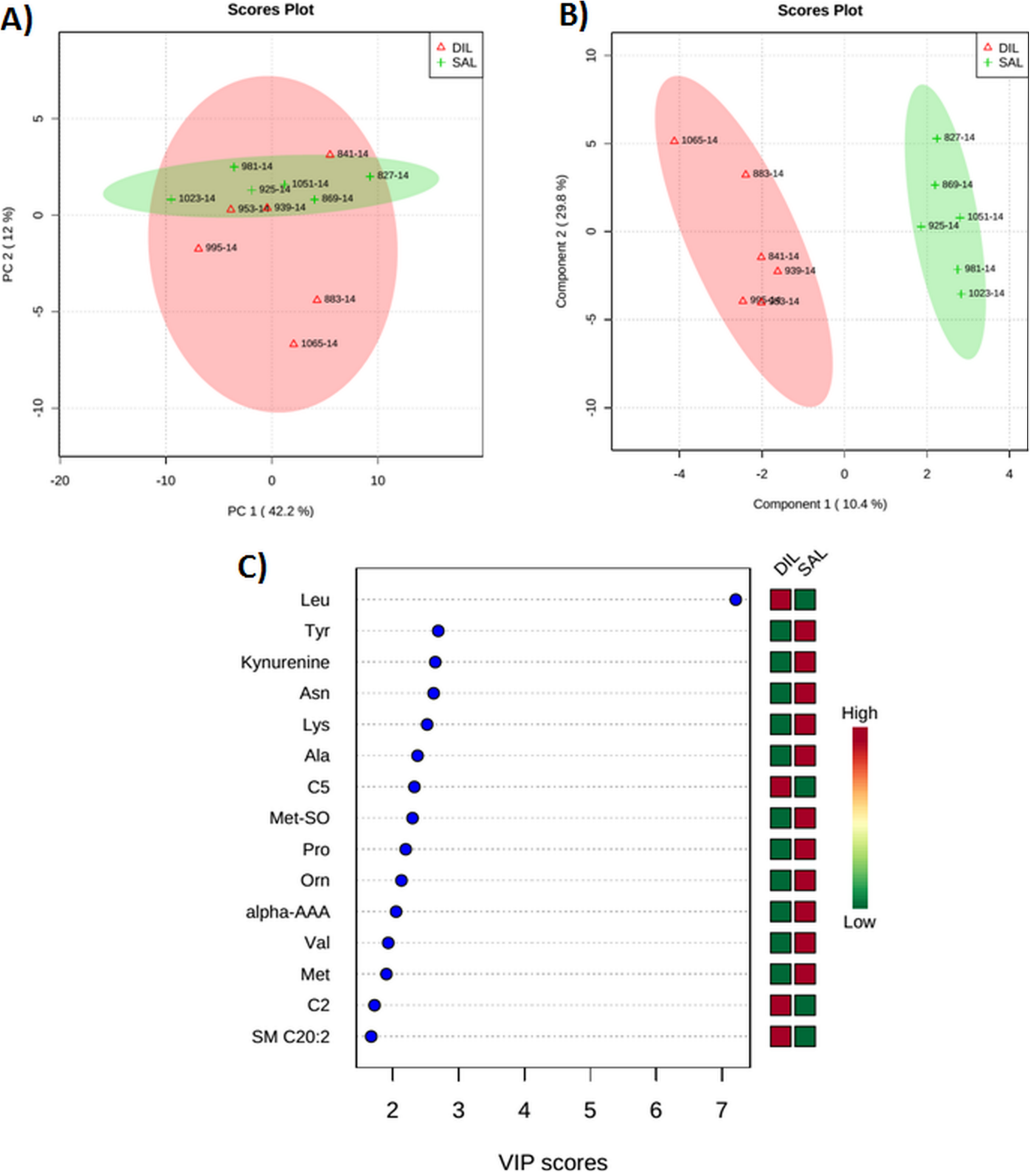
**(A) Score plot of principal component (PC) analysis of dairy cows at 120 min after duodenal bolus infusions of leucine (DIL) as compared with saline (SAL). (B) Partial least squares-discriminant analysis showing 2 clusters for DIL and SAL groups and (C) metabolites ranked by variable importance in projection (VIP).** The numbers in the score plots represent the code numbers of the individual animals.

When DIL cows were compared with SAL cows at 120 min after the infusion, the PCA and PLS-DA plots showed a clear separation (Fig 7A and 7B). In this case, 5 blood plasma metabolites including Leu, Tyr, kynurenine, Asn, and Lys with VIP scores of 7.2, 2.7, 2.6, 2.6, and 2.5 contributed most significantly to the separation between the 2 groups (Fig 7C). All of these metabolites, except for Leu were decreased in the DIL cows as compared with the DIG cows.

When DIG cows were compared with SAL cows at 120 min after the infusion, the multivariate analysis revealed a separation between the 2 groups (S5A and S5B Fig). Five metabolites including Met-SO, Ile, Lys, Orn, and Tyr with corresponding VIP scores of 6.4, 2.9, 2.9, 2.7, and 2.4 contributed most significantly to the observed separation (S5C Fig). All of these metabolites were decreased in the DIG cows as compared with the SAL cows.

## Discussion

As to whether Leu may stimulate insulin release in dairy cows was unknown. For testing this, we selected a bolus rather than a continuous infusion of Leu. The bolus infusion was associated with a rapid increase to supraphysiological (pharmacological) concentrations until a plateau was reached, thus being more promising for a “proof of principle” of the potential insulinotropic effect of Leu in dairy cows. A continuous infusion provides a steady state concentration of the agent and may reduce the likelihood of artefacts; however, a stimulation of insulin release is likely less pronounced as compared to a bolus infusion of the agent. For another insulinotropic agent, for gastric inhibitory polypeptide (GIP), it was shown that stimulation of insulin secretion is stronger after its bolus administration than during continuous infusion [32]. Nevertheless, a bolus infusion of Leu certainly does not reflect daily feeding patterns, and dairy cows may respond differently to an equivalent amount of Leu to be fed continuously.

As expected, none of the variables related to animal performance in this study was affected by the treatments. However, further studies including continuous duodenal infusions of the treatments may provide additional data concerning the potential effects of the treatments on the animal performance. Duodenal infusion of Leu resulted in substantially increased plasma concentrations of Leu in DIL compared to other groups. This observation is in line with findings from other studies also demonstrating elevated plasma concentration of Leu in ruminant animals when it was directly infused into the abomasum [33] or the duodenum [9,34]. The elevation of plasma Leu concentrations after the infusion was associated with a significant decrease in the plasma concentrations of Ile, Val, and Gly starting from 120 min after the infusion and of Ala starting from 40 min after the infusion. Also, mean concentrations of Lys, Met, Phe, Pro, Ser, Tau, Thr, and Asn across all time-points in DIL were lower than in the groups. These results suggest that Leu may stimulate BCAA and other AA influx into the cells from the circulation, leading to an increase in the availability of AA for protein synthesis and/or oxidation in cells. There are many different AA transporter systems that are expressed in a tissue specific manner. The BCAA and aromatic AA including Tyr, Tryptophan (Trp), and Phe share the same AA transport system across the plasma membrane, known as system-L AA transporters (LAT). The LAT system is a Na+-independent AA transporter that mediates transport of other neutral AA, including several essential AA [13,35]. The role of LAT in the regulation of Leu-induced alterations in the plasma AA profile has been recently demonstrated in mice [13]. They found that the elevation of plasma Leu concentration lead to a decrease in the plasma concentrations of Ile, Val, Met, Phe, and Tyr; and that LAT is involved in the mechanism of Leu action. Interestingly, in the current study, the Leu-induced decreases in the plasma AA concentrations occurred 50–60 min after the peak of plasma Leu concentrations, except in case of Ala, which occurred even earlier than the Leu peak. This may suggest that there is a lag time in the action of Leu in reducing the plasma concentrations of BCAA and other AA. It is probable that the elevated cellular Leu concentration, but not that of plasma, is responsible for triggering other AA uptake into cells from the circulation through activation of AA transporters [13]. In the current study, it is also likely that interaction (competition) among AA for uptake has occurred after the bolus duodenal infusion of Leu, and that the reduction of several AA concentrations in the plasma might also be a consequence of less AA uptake in the small intestine that warrant further investigation.

One of the unique features of BCAA metabolism in mammals is its tissue specificity: most essential AA are degraded in the liver, whereas BCAA are removed by the liver (first-pass hepatic catabolism) to a lesser extent than most other AA, and are mostly metabolized in extra-hepatic tissues, in particular skeletal muscle. Another unusual feature of BCAA metabolism is that the first two enzymes of their catabolic disposal, the aminotransferase and the flux-generating dehydrogenase (BCKDH), are common to the three AA [36]. As a consequence, excess dietary Leu in pigs was associated with the stimulation of BCKDH activity, leading to the degradation of not only Leu but also Ile and Val [37,38].

It has been proposed that Leu stimulates insulin secretion through its mitochondrial oxidative decarboxylation as well as by allosterically activating glutamate dehydrogenase in the pancreatic β-cell [14]. It seems that stimulation of mitochondrial activity in the pancreatic β-cell depends on both the generation of acetyl-CoA and α-ketoglutarate [39], resulting in an increased ratio of intracellular ATP to ADP. This leads to the closing of ATP-sensitive K+channels, resulting in plasma membrane depolarization, influx of extracellular Ca2+, and consequently activation of exocytosis of insulin granules from pancreatic β-cells [14,40]. However, in the current study, a single-dose duodenal infusion of Leu did not elicit an apparent insulin response. Supplying intravenously 18% of the daily intake of Leu did not alter the short-term response in plasma insulin concentrations of lactating sows, though the expected increase in the plasma Leu concentrations was observed after the infusion [41]. Possibly, the pancreatic insulin secretion in response to the Leu might have been different in a long-term infusion of Leu. However, a long-term (14 days) duodenal infusion with 0, 3, 6, 9 g/day Leu did not affect plasma insulin concentration, whereas a short-term (10 h) infusion of Leu at the same infusion rate linearly increased insulin secretion in goats [42]. Thus, the results indicate that Leu-induced alterations in the plasma concentrations of BCAA and other AA observed in the present study were likely independent of insulin. Glucagon, secreted from pancreatic α-cells, also responds to both glucose and AA, and that its secretion is stimulated by AA. The short-term effect of glucagon to regulate AA catabolism is through activating AA transporters particularly that for Ala, leading to increased AA uptake by the liver [43]. Interestingly, in the present study, the decrease in the plasma concentrations of Ala was started earlier than other AA, speculatively mediated by the action of glucagon, though no marked changes in the plasma glucagon concentrations were observed. The peripheral concentrations of glucagon may not be equivalent to the total glucagon secreted by the pancreas and reaching the liver through the portal vein [43], so that there may be some increase in the glucagon secretion that was not detected by the changes in the peripheral plasma.

We used a metabolomics approach to gain deeper views into the actual metabolic state of the animals in the time of peak concentrations of plasma Leu, glucose, and insulin (50 min) and of starting the changes in the plasma BCAA and other AA concentrations (120 min). In mammals, Trp is metabolized through 2 metabolic pathways including biosynthesis of the neurotransmitter serotonin and the kynurenine pathway. The kynurenine pathway leading to the production of nicotinamide adenosine dinucleotide (NAD) accounts for 95% of total body Trp metabolism [44]. Kynurenine is the first stable intermediate that is formed in the kynurenine pathway. In the present study, kynurenine, which contributed significantly to the observed separation between the treatment groups, was decreased in the DIL cows as compared with the DIG and SAL cows at both time-points. The effects of high Leu-diet on Trp oxidation along the kynurenine pathway were documented in laboratory animal and in human studies [45]. Interestingly, in the present study, the lower plasma kynurenine was accompanied with the lower plasma Trp in the DIL cows as compared with the DIG and SAL cows at 50 min after the infusion.

Methionine sulfoxide is the oxidized form of Met which is produced through oxidation of the sulfur of Met [46], occurring under both physiological and pathophysiological conditions [47]. The sulfur-containing AA, Met and Cys, are quite readily oxidized as compared with the other AA. Nevertheless, in contrast to other AA, Met-SO can be reduced through NAD(P)H-dependent enzymatic action, by methionine sulfoxide reductases [48]. Therefore, Met-SO might be considered as a biomarker for oxidative stress in vivo [47,49], in particular for oxidative protein damage [50]. In the present study, plasma Met-SO was decreased in the DIL cows as compared with the SAL cows at both time-points. As discussed, the elevation of plasma Leu concentrations after the infusion was associated with a decrease in the mean concentrations of Met in the plasma, probably due to Leu stimulation of Met uptake by the tissues such as skeletal muscle or mammary gland. Thus, the more Met is taken up by tissues, the less Met is retained in the circulation to be oxidized to Met-SO. However, at 50 and 120 min, plasma Met-SO changed inversely in the DIL cows as compared with the DIG cows; a decrease at 50 min and then an increase at 120 min after the infusion were noted. The results suggest that Leu-induced decreases in the plasma AA might not necessarily indicate increased protein synthesis in the body.

Carnitine transports the activated fatty acids from the cytosol into the mitochondrion via their corresponding carnitine ester [51]. Acetylcarnitine, as the shortest acylcarnitine, facilitates the movement of acetyl CoA into the matrices of the mitochondria. Furthermore, in the mitochondria, carnitine acetyl-CoA transferase catalyzes the conversion of acetyl-CoA to C2, a membrane permeable metabolite which facilitate mitochondrial efflux of excess acetyl-CoA [52]. In the present study, plasma C2 was elevated in the DIL cows as compared with the DIG cows at 50 and 120 min after the infusion which may point to an increase in the mitochondrial oxidation of the ketogenic AA including Leu and Lys. Long-chain acylcarnitines have a crucial role in the mitochondrial oxidation of long-chain fatty acids. However, short-chain acylcarnitines are involved in AA metabolism, mainly in the oxidation of BCAA [53]. Plasma concentrations of C3-OH and C3:1 at 50 min and C5 at 120 min after the infusion were elevated in the DIL cows as compared with the SAL cows. Moreover, plasma C5:1-DC (at 50 min), C5, C5:1-DC (at 120 min) were greater in DIL than DIG. We did neither measure AA oxidation nor estimated whole-body protein synthesis in the present study; however, the results indicate accelerated AA oxidation in response to the Leu-infusion.

In the present study, all of the 5 blood plasma metabolites (except for hexose at 50 min) that contributed most significantly to the observed separation between the DIG and SAL at both time points were AA including BCAA that were all decreased in the DIG cows as compared with the SAL cows. Furthermore, the duodenal infusion of glucose expectedly resulted in increased plasma insulin concentrations, suggesting that the stimulatory effect of glucose infusion on AA partitioning may be mediated, at least in part by insulin, as already demonstrated in dairy cows using hyperinsulinemic-euglycemic clamps [54,55]. Interestingly, the response to insulin in terms of protein synthesis in neonatal pigs is specific for skeletal muscle as protein synthesis in the visceral tissues does not respond to insulin infusion [56,57]. However, based on our data we cannot draw conclusions whether insulin acts in the same manner in dairy cows. Glucose infusions were associated with the decrease in plasma concentrations of essential AA, primarily the BCAA [58–60] and that glucose infusion led to partitioning AA toward peripheral, extra-mammary tissues, such as skeletal muscle [61,62] or probably adipose tissue in case of BCAA [60].

In conclusion, a single-dose duodenal infusion of Leu lead to the expected increase in plasma Leu concentrations, but did not alter the plasma insulin; nevertheless, the Leu-induced changes in the plasma AA patterns indicate that Leu may stimulate AA uptake by the tissues through mechanisms yet to be elucidated. The broad metabolite profiling of DIL cows versus DIG and SAL cows, indicates that the Leu infusion affected multiple intermediary metabolic pathways, probably reflecting, at least in parts, increased AA oxidation and energy metabolism.

## Supporting information

S1 Dataset Raw data of study. (XLSX)

S1 Table Crude protein and amino acid contents (% as is) of forages, concentrates, and partial mixed ration (PMR). (PDF)

S1 Fig Volcano plot visualizing plasma metabolites that differ between treatments. (PDF)

S2 Fig Volcano plot visualizing plasma metabolites that differ between treatments at 50 (upper graph) and 120 (lower graph) min after duodenal bolus infusions of leucine as compared with saline in dairy cows. (PDF)

S3 Fig Volcano plot visualizing plasma metabolites that differ between treatments at 50 (upper graph) and 120 (lower graph) min after duodenal bolus infusions of glucose as compared with saline in dairy cows. (PDF)

S4 Fig (A) Score plot of principal component (PC) analysis of dairy cows at 50 min after duodenal bolus infusions of glucose (DIG) as compared with saline (SAL). (B) Partial least squares-discriminant analysis showing 2 clusters for DIL and SAL groups and (C) metabolites ranked by variable importance in projection (VIP).
(PDF)

S5 Fig (A) Score plot of principal component (PC) analysis of dairy cows at 120 min after duodenal bolus infusions of glucose (DIG) as compared with saline (SAL). (B) Partial least squares-discriminant analysis showing 2 clusters for DIL and SAL groups and (C) metabolites ranked by variable importance in projection (VIP).
(PDF)
