# Supplementary material for: Plasma amino acids and metabolic profiling of dairy cows in response to a bolus duodenal infusion of leucine
Source: PLoS One. 2017 Apr 28;12(4):e0176647. doi: 10.1371/journal.pone.0176647 (PMC5409510; doi:10.1371/journal.pone.0176647)
Supplement: S1 Table — (PDF) [file pone.0176647.s002.pdf]

S1 Table Crude protein and amino acid contents (% as is) of forages, concentrates, and partial mixed ration (PMR)

Item	Corn silage	Grass silage	Concentrate[1]	PMR	Concentrate[2]
Crude protein	6.35	11.0	17.9	9.93	17.7
Methionine	0.10	0.12	0.28	0.13	0.29
Cystine	0.08	0.07	0.34	0.11	0.34
Methionine+Cystine	0.19	0.18	0.62	0.23	0.62
Lysine	0.15	0.25	0.85	0.26	0.83
Threonine	0.22	0.29	0.67	0.32	0.67
Arginine	0.09	0.15	1.07	0.23	1.04
Isoleucine	0.22	0.42	0.73	0.36	0.72
Leucine	0.62	0.71	1.39	0.74	1.37
Valine	0.31	0.57	0.86	0.48	0.86
Histidine	0.09	0.11	0.45	0.14	0.45
Phenylalanine	0.24	0.45	0.86	0.42	0.84
Glycine	0.25	0.40	0.78	0.39	0.78
Serine	0.24	0.27	0.85	0.33	0.84
Proline	0.52	0.52	1.26	0.63	1.27
Alanine	0.53	0.70	0.81	0.62	0.80
Apartic acid	0.37	0.66	1.51	0.62	1.47
Glutamic acid	0.76	0.67	3.67	1.10	3.61
GABA	0.18	0.64	-	-	-
Total AA (without NH3)	4.98	6.99	16.4	6.86	16.2
Ammonia	0.22	0.27	0.45	0.36	0.45
Total AA	5.20	7.25	16.8	7.22	16.6

[1]The concentrate used in the PMR

[2]The additional concentrate (4 kg/d/cow) offered using an automatic concentrate feeder
