# Supplementary material for: Plasma amino acids and metabolic profiling of dairy cows in response to a bolus duodenal infusion of leucine
Source: PLoS One. 2017 Apr 28;12(4):e0176647. doi: 10.1371/journal.pone.0176647 (PMC5409510; doi:10.1371/journal.pone.0176647)
Supplement: S1 Fig — (PDF) [file pone.0176647.s003.pdf]

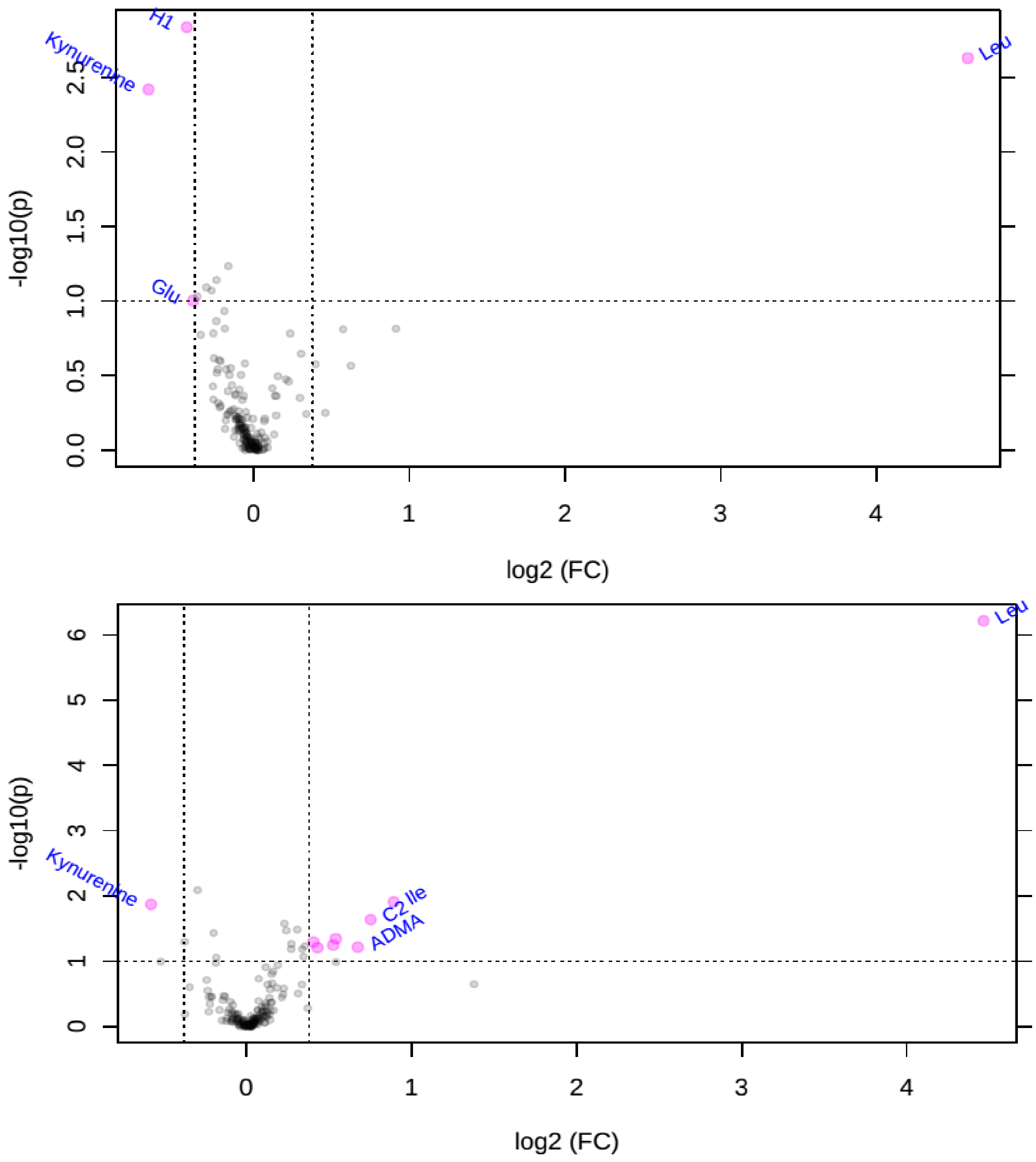

S1 Fig. Volcano plot visualizing plasma metabolites that differ between treatments. Significance (p) versus fold-change (FC) are plotted on the y- and x-axes, respectively. The metabolites are displayed as scattergram, the dashed horizontal line shows the level of significance for the t-tests performed (0.1), the vertical dashed lines indicate the threshold set for FC (1.3) at 50 (upper graph) and 120 (lower graph) min after duodenal bolus infusions of leucine as compared with glucose in dairy cows. The red circles represent metabolites above the thresholds (upper graph: hexose, Leu, kynurenine, Glu; lower graph: Leu, Ile, kynurenine, C2, PC aa C24:0, SDMA, C5, ADMA, C5:1-DC).The name of all metabolites above the thresholds are not displayed in the graphs.
