# Supplementary material for: Plasma amino acids and metabolic profiling of dairy cows in response to a bolus duodenal infusion of leucine
Source: PLoS One. 2017 Apr 28;12(4):e0176647. doi: 10.1371/journal.pone.0176647 (PMC5409510; doi:10.1371/journal.pone.0176647)
Supplement: S2 Fig — (PDF) [file pone.0176647.s004.pdf]

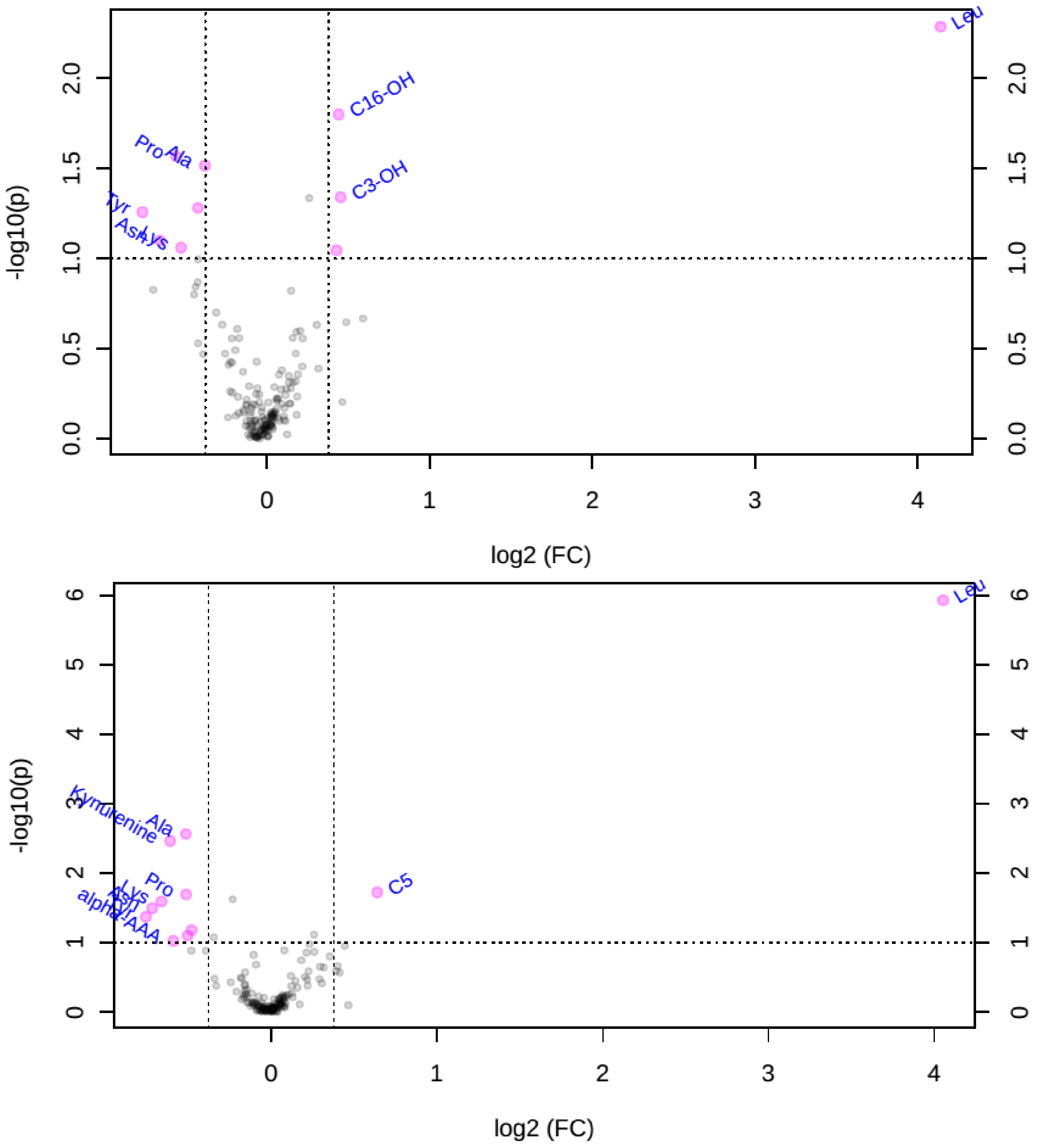

S2 Fig. Volcano plot visualizing plasma metabolites that differ between treatments at 50 (upper graph) and 120 (lower graph) min after duodenal bolus infusions of leucine as compared with saline in dairy cows. The red circles represent metabolites above the threshold (upper graph: Leu, C16-OH, Pro, Ala, C3-OH, Trp, Tyr, Asn, Lys, C3:1; lower graph: Leu, Ala, kynurenine, C5, Pro, Lys, Asn, Tyr, Val, ornithine, alpha-aminoadipic acid). The names of all metabolites above the thresholds are not displayed in the graphs. Further characteristics of volcano plots in general are given in S1 Fig.
