# Supplementary material for: Plasma amino acids and metabolic profiling of dairy cows in response to a bolus duodenal infusion of leucine
Source: PLoS One. 2017 Apr 28;12(4):e0176647. doi: 10.1371/journal.pone.0176647 (PMC5409510; doi:10.1371/journal.pone.0176647)
Supplement: S4 Fig — (A) Score plot of principal component (PC) analysis of dairy cows at 50 min after duodenal bolus infusions of glucose (DIG) as compared with saline (SAL). (B) Partial least squares-discriminant analysis showing 2 clusters for DIL and SAL groups and (C) metabolites ranked by variable importance in projection (VIP). (PDF) [file pone.0176647.s006.pdf]

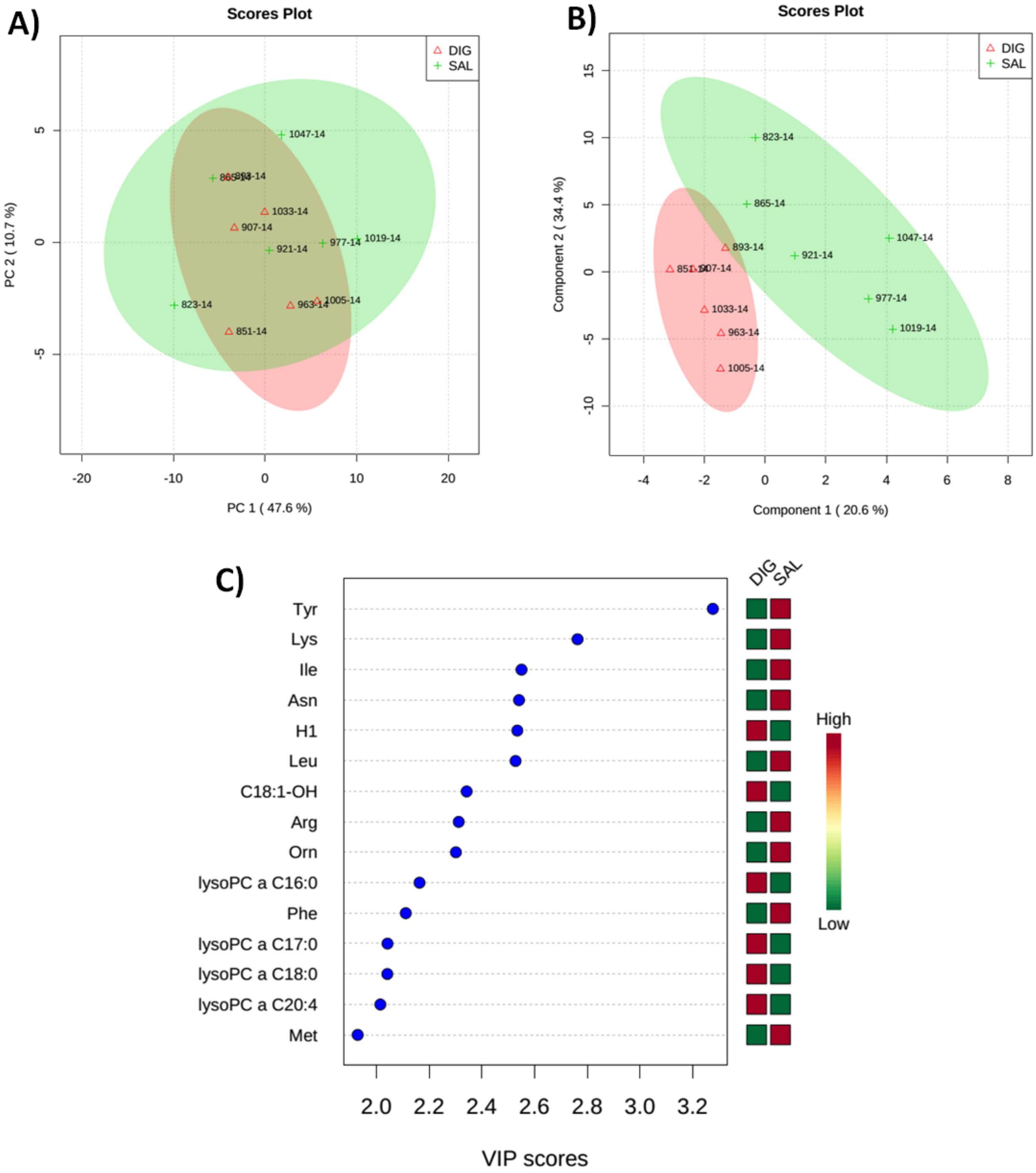

S4 Fig. (A) Score plot of principal component (PC) analysis of dairy cows at 50 min after duodenal bolus infusions of glucose (DIG) as compared with saline (SAL). (B) Partial least squares-discriminant analysis showing 2 clusters for DIL and SAL groups and (C) metabolites ranked by variable importance in projection (VIP). The numbers in the score plots represent the code numbers of the individual animals.
